# Study on minerals status of dairy cows and their supplementation through area specific mineral mixture in the state of Jharkhand

**DOI:** 10.1186/s40781-016-0124-2

**Published:** 2016-12-19

**Authors:** B. M. Bhanderi, Ajay Goswami, M. R. Garg, Saikat Samanta

**Affiliations:** 1 Animal Nutrition Group, National Dairy Development Board (NDDB), Anand, 388001 Gujarat India; 2 Head (Productivity Enhancement), Jharkhand Milk Federation, Ranchi, Jharkhand State India

**Keywords:** Calcium, Phosphorus, Copper, Zinc, Feeds, Forage, Dairy cows

## Abstract

**Background:**

Deficiency of macro and micro-minerals in the ration of dairy cows adversely affects growth, milk production and reproduction efficiency. It is essential to examine mineral concentrations in feeds offered to dairy cows in practical farms.

**Methods:**

Two villages from each taluka were selected at random for taking representative samples of feeds, forages and hair. Within the village, help was sought from village milk producers and district animal husbandry officer for identification of 4 to 5 farmers and collection of representative samples. All the samples were processed and analyzed for chemical composition as well as major macro and micro-minerals, using Inductively Coupled Plasma-Optical Emission Spectrometer.

**Results:**

Ca content in wheat straw (0.29%), crushed maize (0.02%) and wheat bran (0.12%) was found to be below the critical level (0.30%). The P content in concentrate ingredients was high (0.26–0.96%), but low in dry roughages (0.06–0.12%). Cereal straws (0.14%) and grains (0.12%) were deficient in Mg. Feeds and forages were found to be adequate in K (1.50%). Cereals straws were found to be deficient in S (0.11%). Greens were good source of Cu (12.02 ppm). Wheat straw was found to be low in Zn (18 ppm), but high in Mn (225 ppm) and Fe (509 ppm). Local grasses and azolla green were found to be rich source of Co (>1.00 ppm). Se (0.63 ppm) was present in appreciable quantities in most of the feedstuffs.

**Conclusions:**

From the present study, it was apparent that the feeds and forages available in the state of Jharkhand may not meet the requirements for Ca, P, Mg, Cu, Zn and Co in order to sustain a milk production of ~10 kg/day. Therefore, it is necessary to supplement these deficient minerals through area specific mineral mixture in the ration of dairy cows for improving productivity and reproduction efficiency.

## Background

Dietary nutrition plays a significant role in any livestock development programme and the optimum expression of genetic potential for milk production in dairy cows depend on adequate supply of nutrients. Micronutrients, particularly the mineral elements are considered to be inevitable for the normal metabolic and physiological processes of animal systems. The importance of minerals in regulating biological systems, growth, production and reproduction is well documented [1], however, livestock in India do not receive mineral/vitamin supplements except for common salt and calcite powder [2]. Hence, dairy cows depend on forages for their mineral requirements [3]. Garg et al. [4] and Bhanderi et al. [5] reported high incidences of forage and blood serum samples below the critical levels for Cu and Zn. Miles and McDowell [6] demonstrated deficiency of Cu and P in the forage samples collected from the pasture. Soils from all over country have been being depleted for Cu, Zn, P and S in soil, plants and dairy cows [7]. The quantity of minerals, thus, present in forages may not be sufficient for optimum growth, milk yield and reproduction when those were fed to dairy cows [3]. In order to avoid macro and micro-minerals deficiency in the ration, a study on the assessment of mineral status of Holstein Friesian crossbred cows was undertaken in the state of Jharkhand.

## Methods

### Sampling procedures

Two villages from each taluka were selected at random for taking representative samples of feeds, forage and hair. Total area of Jharkhand state is 79,710 sq km distributed in to 24 districts, 211 blocks and 32,620 villages. The district is having annual rainfall of 1,400 mm, latitude of 23^0^35^’^ N and longitude of 85^0^33’ E. Atmospheric temperature ranges from 5 to 45 °C during different seasons. In this study, two dimensional survey methods was adopted to map relevant mineral elements, by collecting feeds and forage samples from the representative villages, according to random sampling design based on conceptual landscape units [8]. Within the village, help was sought from village milk producers and district animal husbandry officer for identification of 4 to 5 farmers and collection of representative samples. The recorded parameters were number of livestock, land area, irrigation facilities, forage, other crops being grown, feeding practices followed, quantity of feeds, forage, dry roughage, concentrate feeds and mineral mixture offered to dairy cows. Representative samples of feeds, wet forages and dry roughages were collected from northern, eastern, western and southern directions of the selected villages. Further information regarding the amount and types of feeds and forages being offered to the dairy cows, actual rate of daily feed intake, number of milking cows and milk yield were collected from individual farmer. Daily feed intakes were monitored through the INAPH software. INAPH or *Information Network for Animal Productivity and Health*, a windows based Internet linked application, developed by National Dairy Development Board of India to assess the prevailing status of the nutrient provision to the animal against the animal’s nutrient requirements [9]. Both these sets of information are used to work out a least cost ration with the available feed resources and an area specific mineral mixture [10]. Total intake was compared against the requirements on dry matter basis [11], so as to identify quantitative deficiency, adequacy or even excess. With the help of INAPH software, status of metabolizable energy, protein intake and mineral status of dairy cows reared under field conditions was assessed [9].

### Sample preparation and analytical methods

Weekly basis composite samples of wet forages, cereal straws/dry forages, concentrate feed ingredients and the compound cattle feed (concentrate mixture) were collected from all over the surveyed area. Surveyed area used for the places/villages in the district from where representative samples of feed, wet forage, dry roughage and hair samples were collected. Wet forage samples were dried in hot air oven at 100 °C for 24 h and subsequently ground (1 mm). Ground samples of concentrate and forages were stored in airtight bags until analysis. The samples of feeds, wet forages and dry roughages were analyzed for crude protein (CP), ether extract (EE), crude fibre (CF) and acid insoluble ash (AIA) as per AOAC [12] and for neutral detergent fibre and acid detergent fibre as per Van Soest et al. [13]. All the samples were also analyzed for calcium (Ca), phosphorus (P), magnesium (Mg), sulphur (S), sodium (Na), potassium (K), copper (Cu), zinc (Zn), manganese (Mn), iron (Fe), cobalt (Co), selenium (Se) and molybdenum (Mo), using Inductively Coupled Plasma-Optical Emission Spectrometer (Perkin-Elmer, OPTIMA-3300 RL).

The word “critical” is used in this article to note a concentration in feedstuffs below (or above with excesses) what is considered the requirement for dairy cow [14]. This assumes the expected consumption as estimated by the NRC [11]. Total grams/milligrams of minerals consumed per day determine the true adequacy of a mineral, not the forage concentration [15].

### Statistical analysis

The data were analyzed statistically as per the Snedecor and Cochran [16], with the help of SPSS package programme (SPSS 9.00 software for Windows, SPSS Inc., Chicago, IL).

## Results and discussion

Crop residues were found to be the main source of dry roughages in the ration of dairy cows. It was noticed that some of the milk producers fed cultivated forages like, maize (*Zea mays*), *jowar* green (*Sorghum bicolor*) etc. Some milk producers offered crushed maize, crushed wheat alone or their mixture. Feeding mustard oil cake, wheat bran, linseed cake and maize germ cake was also observed in some parts of state. Those milk producers, who didn’t feed concentrate ingredients, were feeding compound cattle feed depending on the level of milk production. The use of common salt and mineral mixture supplementation was not a common practice in the surveyed area, except for therapeutic purpose on prescription by veterinary officer.

### Chemical composition of feeds and forages

The feed and forage samples collected from surveyed area were analyzed for chemical composition (Table 1). Mustard oil cake was a good source of protein (37.98% crude protein), whereas, linseed meal contained 32.84% crude protein (CP). Amongst wet forage azolla (22.18%) had the highest CP content followed by maize (7.24%) and para grass (5.35%). Cereal straws/dry forages were low in CP content, except gotars of Bengal gram (*Cicer arietinum*), arhar/pigeon pea (*Cajanus cajan*) and urd (*Phaseolus mungo*). Neutral detergent fibre (NDF) and acid detergent fibre (ADF) content was found to be highest in dry roughages. The data on proximate composition of the feedstuffs are in agreement [17–19].

**Table 1:** Chemical composition of feed and forages in Jharkhand state (on DM basis)

Feed item	CP (%)	EE (%)	CF (%)	NDF (%)	ADF (%)	AIA (%)
Grains/seeds						
Crushed wheat (4), *Triticum aestivum*	11.95 ± 0.43	1.98 ± 0.09	1.25 ± 0.07	14.82 ± 0.67	5.63 ± 0.11	0.23 ± 0.04
Crushed maize (5), *Zea mays*	8.65 ± 0.12	3.48 ± 0.09	1.92 ± 0.08	15.62 ± 0.12	4.59 ± 0.04	0.26 ± 0.05
Brans/cakes/chunnies						
Wheat bran (5), *Triticum aestivum*	15.08 ± 0.29	2.08 ± 0.08	16.78 ± 0.62	62.38 ± 0.98	18.93 ± 0.68	3.48 ± 0.11
Mustard oil cake (6), *Brassica campestris*	37.98 ± 1.25	6.77 ± 0.34	7.18 ± 0.23	26.66 ± 0.69	17.84 ± 0.39	2.46 ± 0.18
Linseed meal (4*), Linum usitatissimum*	32.84 ± 0.86	0.62 ± 0.08	9.5 ± 0.34	24.18 ± 0.27	18.67 ± 0.19	2.05 ± 0.18
Sesame cake (3), *Sesamum indicum*	29.80 ± 1.13	8.46 ± 0.68	8.90 ± 0.91	27.44 ± 0.86	16.88 ± 0.48	3.11 ± 0.16
Gram chunni (3), *Cicer arietinum*	18.80 ± 1.24	3.56 ± 0.32	11.38 ± 0.23	32.18 ± 2.48	18.68 ± 0.93	0.78 ± 0.11
Masoor chunni + arhar chunni (4*), Lens culinaris + Cajanus cajan*	20.85 ± 0.67	1.90 ± 0.34	12.88 ± 0.22	28.38 ± 2.78	17.82 ± 0.82	1.86 ± 0.18
Green fodder/grasses						
Maize green (5), *Zea mays*	7.24 ± 0.11	1.25 ± 0.08	27.44 ± 0.18	52.68 ± 3.22	33.17 ± 1.23	2.36 ± 0.11
Para grass (4*), Brachiaria mutica*	5.35 ± 0.16	1.65 ± 0.11	30.44 ± 0.46	64.89 ± 3.88	31.33 ± 1.44	2.90 ± 0.19
Local green grasses (6)	4.65 ± 0.09	1.93 ± 0.12	34.68 ± 0.23	58.66 ± 2.88	29.89 ± 2.08	3.11 ± 0.18
Azolla green (6), *Azolla pinnata*	22.18 ± 1.45	3.89 ± 0.48	12.7 ± 0.17	36.78 ± 1.88	23.18 ± 1.18	2.56 ± 0.13
Straws/gotars						
Wheat straw (6), *Triticum aestivum*	2.15 ± 0.11	0.69 ± 0.06	38.44 ± 2.34	78.58 ± 2.33	56.12 ± 3.12	5.44 ± 0.21
Paddy straw (5), *Oryza sativa*	3.44 ± 0.18	1.11 ± 0.15	42.32 ± 1.89	68.75 ± 2.44	44.78 ± 3.19	5.18 ± 0.23
Gram gotar (4); *Cicer arietinum*	8.11 ± 0.11	2.01 ± 0.19	34.67 ± 1.88	54.89 ± 3.19	38.67 ± 2.18	3.90 ± 0.21
Arhar gotar (6), *Cajanus cajan*	8.75 ± 0.19	1.89 ± 0.18	33.88 ± 2.15	56.14 ± 2.28	40.18 ± 3.88	4.18 ± 0.28
Urd gotar (4), *Phaseolus mungo*	9.44 ± 0.34	1.44 ± 0.12	29.44 ± 1.09	58.33 ± 1.90	42.23 ± 2.19	4.88 ± 0.31

Figures in the parentheses indicate no. of samples analysed

### Nutritional status of lactating dairy cows

In order of priority, available good quality feed resources are first allocated to lactating dairy cows followed by dry pregnant, dry, heifers, growing calves and non-productive cows. In the surveyed area our observation indicates that metabolizable energy were in excess in the ration of more than 65% of cows, crude protein was deficient in the diet of more than 85% of dairy cows, Ca and P were deficient in the ration of about 70% of the cows because of inadequate mineral mixture supplementation. Milk producers in most of the developing countries often do not feed adequate quantities of mineral mixture to their dairy cows due to non-availability, lack of knowledge on the benefits of feeding mineral mixtures [20]. In view of this, there is an urgent need to popularize supplementation of mineral mixture in the ration of dairy cows for improving production and reproduction efficiency [21]. In developing countries, Garg and Sherasia [22] reported that daily milk production of dairy cows is low, as compared to developed nations. Similarly, Garg et al. [23] reported that age at first calving and inter-calving intervals is higher in Zebu dairy cows, affecting life time productivity in developing countries.

### Macro-minerals profile of feeds and forages

The straws of wheat and paddy were the main roughage sources in the surveyed area (Tables 2 and 3). The average Ca content ranged from 0.29 to 1.17% in roughages as compared to 0.02 to 0.68% in concentrate feed ingredients. These findings are similar to the findings of Ramana et al. [24]. The similar findings were also reported by Udar et al. [25] and Bhanderi et al. [26]. P content in concentrates (0.17 to 0.96%) was higher than dry roughages (0.06 to 0.13%). Crushed grains were low in Mg as compared to cakes (Table 3). S content was found below critical level (<0.20%) in most of the straws and crushed grain [27]. Higher K level in wet forages may be due to its selective uptake from the soil and regular application of potash fertilizer in the soil [2]. Na content was low in some of the feedstuffs (Tables 2 and 3).

**Table 2:** Macro-minerals content in dry and wet forages (on DM basis)

Particular	Ca	P	Mg	S	K	Na
Critical level*	<0.30%	<0.25%	<0.20	<0.20%	<0.9%	<0.06%
Wheat straw (69), *Triticum aestivum*	0.29 ± 0.02	0.06 ± 0.00	0.12 ± 0.01	0.12 ± 0.01	1.43 ± 0.08	0.04 ± 0.01
Paddy straw (123), *Oryza sativa*	0.36 ± 0.01	0.08 ± 0.00	0.17 ± 0.00	0.11 ± 0.00	1.71 ± 0.04	0.12 ± 0.01
Gram straw (4), *Cicer arietinum*	1.04 ± 0.02	0.11 ± 0.01	0.26 ± 0.01	0.21 ± 0.01	2.05 ± 0.09	0.07 ± 0.01
Masoor straw (3), *Lens culinaris*	1.17 ± 0.03	0.11 ± 0.01	0.23 ± 0.02	0.15 ± 0.02	2.01 ± 0.04	0.04 ± 0.01
Arhar straw (6), *Cajanus cajan*	1.12 ± 0.03	0.13 ± 0.00	0.27 ± 0.01	0.17 ± 0.00	1.45 ± 0.1	0.03 ± 0.01
Urd straw (4), *Phaseolus mungo*	0.94 ± 0.02	0.12 ± 0.31	1.69 ± 0.46	2.08 ± 0.68	3.70 ± 0.81	2.51 ± 0.88
Maize green (22), *Zea mays*	0.58 ± 0.05	0.33 ± 0.03	0.35 ± 0.02	0.26 ± 0.02	3.33 ± 0.31	0.03 ± 0.01
Sudan grass (11), *Sorghum sudanense*	0.58 ± 0.05	0.25 ± 0.01	0.32 ± 0.02	0.19 ± 0.00	2.28 ± 0.06	0.06 ± 0.00
Cowpea green (4), *Vigna sinensis*	2.01 ± 0.2	0.33 ± 0.03	0.46 ± 0.04	0.34 ± 0.01	2.27 ± 0.16	0.03 ± 0.01
Azolla green (12), *Azolla pinnata*	1.50 ± 0.08	0.84 ± 0.23	0.41 ± 0.07	0.40 ± 0.04	2.84 ± 0.46	0.70 ± 0.13
Bajri green (4), *Pennisetum typhoides*	0.59 ± 0.06	0.45 ± 0.04	0.30 ± 0.01	0.21 ± 0.02	2.59 ± 0.42	0.01 ± 0.01
Para grass (4), *Brachiaria mutica*	0.63 ± 0.07	0.34 ± 0.02	0.19 ± 0.02	0.22 ± 0.01	5.05 ± 0.76	0.01 ± 0.02
Local green grasses (48)	0.88 ± 0.07	0.33 ± 0.02	0.41 ± 0.03	0.38 ± 0.02	3.16 ± 0.15	0.12 ± 0.02

Figures in the parentheses indicate no. of samples analysed

*Concentrations below which are low or considered to be deficient (McDowell et al., 1993), based on requirements for cattle [11]

**Table 3:** Macro-minerals content in concentrate feed ingredients (on DM basis)

Particular	Ca	P	Mg	S	K	Na
Critical level*	<0.30%	<0.25%	<0.20%	<0.20%	<0.9%	<0.06%
Crushed maize (25), *Zea mays*	0.02 ± 0.00	0.26 ± 0.01	0.10 ± 0.00	0.11 ± 0.00	0.37 ± 0.01	0.01 ± 0.00
Wheat bran (81), *Triticum aestivum*	0.12 ± 0.02	0.84 ± 0.03	0.33 ± 0.01	0.20 ± 0.00	1.06 ± 0.03	0.02 ± 0.00
Mustard oil cake (54), *Brassica campestris*	0.68 ± 0.03	0.96 ± 0.03	0.40 ± 0.02	1.26 ± 0.05	1.12 ± 0.04	0.02 ± 0.00
Cattle feed (21)	0.99 ± 0.1	1.41 ± 0.06	0.78 ± 0.03	0.44 ± 0.03	1.26 ± 0.05	0.65 ± 0.06
Gram chunni (9), *Cicer arieninum*	0.63 ± 0.14	0.25 ± 0.02	0.33 ± 0.08	0.13 ± 0.01	0.73 ± 0.03	0.02 ± 0.00
Rice bran (15), *Oryza sativa*	0.11 ± 0.01	0.46 ± 0.05	0.29 ± 0.02	0.11 ± 0.01	0.61 ± 0.07	0.01 ± 0.00
Maize + wheat mixture (17), *Zea mays + Triticum aestivum*	0.20 ± 0.07	0.36 ± 0.02	0.20 ± 0.03	0.17 ± 0.02	0.61 ± 0.06	0.02 ± 0.01
Crushed wheat (12), *Triticum aestivum*	0.06 ± 0.01	0.31 ± 0.01	0.12 ± 0.00	0.15 ± 0.01	0.41 ± 0.01	0.00 ± 0.00
Wheat grain (5), *Triticum aestivum*	0.04 ± 0.00	0.30 ± 0.01	0.11 ± 0.00	0.17 ± 0.01	0.38 ± 0.01	0.01 ± 0.00
Gram flour (15), *Cicer arietinum*	0.09 ± 0.02	0.29 ± 0.02	0.12 ± 0.01	0.17 ± 0.02	0.74 ± 0.08	0.01 ± 0.00
Kesari dal (3), *Lathyrus sativus*	0.13 ± 0.00	0.28 ± 0.00	0.14 ± 0.00	0.18 ± 0.01	0.73 ± 0.09	0.02 ± 0.00
Linseed cake (6), *Linum usitatissimum*	0.49 ± 0.04	0.58 ± 0.02	0.38 ± 0.01	0.35 ± 0.04	0.92 ± 0.04	0.02 ± 0.00
Maize grain (5), *Zea mays*	0.01 ± 0.00	0.27 ± 0.01	0.10 ± 0.01	0.11 ± 0.01	0.39 ± 0.01	0.01 ± 0.00
Masoor chunni (4), *Lens culinaris*	0.37 ± 0.02	0.21 ± 0.06	0.28 ± 0.02	0.10 ± 0.01	0.50 ± 0.02	0.04 ± 0.00
Masoor + arhar mix chunni (6), *Lens culinaris + Cajanus cajan*	0.43 ± 0.01	0.17 ± 0.03	0.21 ± 0.01	0.12 ± 0.01	0.83 ± 0.15	0.04 ± 0.01
Sesame cake (6), *Sesamum indicum*	0.64 ± 0.18	0.77 ± 0.04	0.45 ± 0.02	0.54 ± 0.06	1.12 ± 0.04	0.05 ± 0.01
Jowar green (2), *Sorghum bicolor*	0.43 ± 0.05	0.29 ± 0.02	0.27 ± 0.02	0.20 ± 0.01	2.01 ± 0.16	0.01 ± 0.00
Wheat flour (7), *Triticum aestivum*	0.06 ± 0.01	0.30 ± 0.01	0.13 ± 0.00	0.18 ± 0.00	0.41 ± 0.02	0.03 ± 0.00
Bengal gram (2), *Cicer arietinum*	0.23 ± 0.02	0.49 ± 0.04	0.18 ± 0.01	0.27 ± 0.02	1.07 ± 0.03	0.03 ± 0.01
Maize cake (2), *Zea mays*	0.07 ± 0.01	0.28 ± 0.02	0.06 ± 0.01	0.24 ± 0.02	0.13 ± 0.01	0.02 ± 0.01

Figures in the parentheses indicate no. of samples analysed

*Concentrations below which are low or considered to be deficient (McDowell et al., 1993), based on requirements for cattle [11]

### Micro-minerals profile of feeds and forages

Cu content was found below the critical level (<8 ppm) in wheat straw, paddy straw and crushed grains (Tables 4 and 5). Zn content was below critical level (<30 ppm) in all the straws except paddy straw [27}. Wet forages and cakes were found to be a better source of Zn as compared to crushed grains (Tables 4 and 5). The Mn levels in the state ranged from 36.88–662.56 ppm in straws, 45.21–254.29 ppm in wet forages and 4.97–200.17 ppm in concentrate ingredients. Average Fe content was 1037 ppm in roughage and 651 ppm in concentrates, showing adequacy of this mineral. Youssef et al. [28] and Yadav et al. [29] reported high Fe levels in forages. Co content in most of the feeds and forages ranged from 0.02 ppm to 1.33 ppm, except in azolla green in which it was as high as 17.67 (Tables 4 and 5). Se and Mo content were adequate in all the feeds and forages. High levels of Mo (>2 ppm) in forages could interfere with Cu metabolism. The Mo levels as estimated in the samples of crop residues were within the safe limit. Most of the feedstuffs contained Mo level within the safe limit and gave Cu:Mo ratio wider than 5.0. Mo has gained more importance recently in animal nutrition, because of its inhibitory role on the other trace elements, particularly Cu. Suttle [30] stated that a Cu:Mo ratio below 2.0 would be expected to cause conditioned Cu deficiency in dairy cows. Mo level at 5 to 6 ppm inhibits Cu storage and produce signs of molybdenosis [31]. Even 2 ppm or less can be toxic, if forage Cu is sufficiently low [32]. In case of ruminants, Mo reacts with sulphur in the rumen and forms mono-, di-, tri- or tetra-thiomolybdates [30], making Cu unavailable for absorption and utilization [33].

**Table 4:** Micro-minerals content in dry and wet forages (on DM basis)

Particular	Cu	Zn	Mn	Fe	Co	Mo	Se
Critical level*	<8 ppm	<30 ppm	<40 ppm	<50 ppm	<0.10 ppm	>6 ppm	<0.2 ppm
Wheat straw (69), *Triticum aestivum*	2.46 ± 0.21	18.00 ± 2.85	225 ± 58.7	509 ± 62	0.32 ± 0.06	0.56 ± 0.03	1.33 ± 0.21
Paddy straw (123), *Oryza sativa*	2.99 ± 0.14	39.79 ± 1.17	663 ± 28.2	851 ± 82	0.73 ± 0.06	0.21 ± 0.02	0.78 ± 0.00
Gram straw (4), *Cicer arietinum*	5.93 ± 0.02	17.20 ± 1.28	75 ± 12.3	1,015 ± 136	0.56 ± 0.06	0.87 ± 0.11	0.64 ± 0.07
Masoor straw (3), *Lens culinaris*	7.01 ± 0.33	20.49 ± 3.33	52 ± 3.1	988 ± 93	0.56 ± 0.04	0.28 ± 0.08	0.36 ± 0.05
Arhar straw (6), *Cajanus cajan*	7.34 ± 0.43	24.08 ± 1.06	75 ± 2.6	1,124 ± 65	0.70 ± 0.05	0.72 ± 0.05	0.54 ± 0.05
Urd straw (4), *Phaseolus mungo*	10.42 ± 0.09	24.97 ± 5.10	31 ± 6.0	209 ± 33	0.11 ± 0.02	0.36 ± 0.06	0.30 ± 0.08
Maize green (22), *Zea mays*	10.22 ± 1.26	37.91 ± 2.07	179 ± 44.1	1,217 ± 189	0.72 ± 0.11	1.96 ± 0.30	0.71 ± 0.10
Sudan grass (11), *Sorghum sudanense*	09.38 ± 1.00	31.36 ± 1.58	241 ± 64.7	1,018 ± 105	0.51 ± 0.07	0.29 ± 0.10	0.15 ± 0.05
Cowpea green (4), *Vigna sinensis*	10.42 ± 1.08	39.43 ± 2.24	135 ± 12.6	1,088 ± 105	0.55 ± 0.05	2.52 ± 0.39	1.05 ± 0.12
Azolla green (12), *Azolla pinnata*	11.38 ± 2.16	131.23 ± 40.55	4,068 ± 826	2,599 ± 43	17.67 ± 2.92	5.06 ± 1.25	0.52 ± 0.10
Bajri green (4), *Pennisetum typhoides*	19.19 ± 1.34	28.46 ± 2.78	45 ± 7.1	465 ± 197	0.25 ± 0.08	1.35 ± 0.10	0.76 ± 0.10
Para grass (4), *Brachiaria mutica*	13.14 ± 0.94	29.29 ± 3.20	55 ± 1.5	426 ± 39	0.26 ± 0.03	0.08 ± 0.47	0.08 ± 0.02
Local green grasses (48)	10.43 ± 0.93	42.83 ± 3.22	254 ± 35.5	1,972 ± 506	1.33 ± 0.38	0.08 ± 0.02	0.48 ± 0.05
Jowar green (4), *Sorghum bicolor*	09.69 ± 1.85	22.77 ± 4.39	115 ± 34.4	548 ± 99	0.35 ± 0.06	0.07 ± 0.00	0.28 ± 0.05

Figures in the parentheses indicate no. of samples analyzed

*Concentrations below which are low or considered to be deficient (McDowell et al., 1993), based on requirements for cattle [11]

**Table 5:** Micro-minerals content in concentrate feed ingredients (on DM basis)

Particular	Cu	Zn	Mn	Fe	Co	Mo	Se
Critical level*	<8 ppm	<30 ppm	<40 ppm	<50 ppm	<0.10 ppm	>6 ppm	<0.2 ppm
Crushed maize (25), *Zea mays*	02.81 ± 0.26	21.31 ± 0.49	11 ± 0.5	185 ± 38	0.10 ± 0.01	1.63 ± 0.07	1.53 ± 0.12
Wheat bran (81), *Triticum aestivum*	11.77 ± 0.46	75.21 ± 2.11	100 ± 6.7	475 ± 62	0.23 ± 0.03	0.46 ± 0.05	1.35 ± 0.16
Mustard oil cake (54), *Brassica campestris*	09.97 ± 0.44	62.32 ± 1.60	70 ± 5.7	1,019 ± 95	0.48 ± 0.05	2.57 ± 0.06	0.60 ± 0.04
Cattle feed (21)	12.71 ± 0.75	124.29 ± 11.08	192 ± 12.5	1,147 ± 120	1.21 ± 0.14	0.95 ± 0.15	0.28 ± 0.04
Gram chunni (9), *Cicer arietinum*	12.25 ± 2.03	29.33 ± 2.12	51 ± 6.2	608 ± 96	0.38 ± 0.04	3.06 ± 0.15	0.02 ± 0.29
Rice bran (15), *Oryza sativa*	16.48 ± 2.04	48.06 ± 5.15	200 ± 14.6	791 ± 76	0.32 ± 0.06	0.45 ± 0.05	0.91 ± 0.14
Maize + wheat mixture (17), *Zea mays + Triticum aestivum*	8.57 ± 0.59	35.77 ± 2.61	43 ± 5.7	713 ± 103	0.41 ± 0.07	0.34 ± 0.33	1.58 ± 0.25
Crushed wheat (12), *Triticum aestivum*	4.17 ± 0.28	29.31 ± 1.66	30 ± 1.7	217 ± 51	0.18 ± 0.03	0.68 ± 0.03	0.77 ± 0.17
Wheat grain (5), *Triticum aestivum*	4.97 ± 0.35	31.16 ± 1.95	47.5 ± 3.2	106 ± 11	0.18 ± 0.03	0.03 ± 0.00	0.68 ± 0.18
Gram flour (15), *Cicer arietinum*	06.89 ± 1.18	30.69 ± 3.07	24 ± 3.2	221 ± 70	0.30 ± 0.05	0.77 ± 2.45	1.03 ± 0.08
Kesari dal (3), *Lathyrus sativus*	6.54 ± 0.38	32.36 ± 1.15	43 ± 4.0	999 ± 82	0.59 ± 0.01	0.70 ± 0.23	0.40 ± 0.11
Linseed cake (6), *Linum usitatissimum*	20.05 ± 1.51	57.82 ± 1.56	70 ± 4.9	1,715 ± 97	1.32 ± 0.08	0.97 ± 0.28	0.64 ± 0.15
Maize grain (5), *Zea mays*	3.60 ± 0.68	20.23 ± 2.65	10 ± 8.6	278 ± 34	0.18 ± 0.05	1.84 ± 0.23	0.21 ± 0.11
Masoor chunni (4), *Lens culinaris*	10.70 ± 0.27	44.67 ± 1.61	76 ± 3.1	1,992 ± 10	1.29 ± 0.03	0.03 ± 0.00	0.68 ± 0.18
Masoor + arhar mix chunni (6), *Lens culinaris + Cajanus cajan*	13.60 ± 0.31	40.97 ± 2.81	27 ± 1.5	388 ± 73	0.30 ± 0.01	0.77 ± 2.45	0.86 ± 0.12
Sesame cake (6), *Sesamum indicum*	21.88 ± 1.04	78.42 ± 1.72	60 ± 2.6	1,098 ± 90	0.61 ± 0.05	0.70 ± 0.23	0.40 ± 0.11
Wheat flour (7), *Triticum aestivum*	5.25 ± 0.04	31.81 ± 0.42	61 ± 12.4	199 ± 17	0.13 ± 0.02	0.97 ± 0.28	0.75 ± 0.26
Bengal gram (2), *Cicer arietinum*	9.91 ± 0.68	48.88 ± 2.65	30 ± 8.6	173 ± 34	0.36 ± 0.05	0.69 ± 0.23	0.21 ± 0.11
Maize germ cake (2), *Zea mays*	4.04 ± 0.38	24.61 ± 3.65	5 ± 0.6	158 ± 24	0.02 ± 0.00	0.19 ± 0.03	0.01 ± 0.00

Figures in the parentheses indicate no. of samples analyzed

*Concentrations below which are low or considered to be deficient (McDowell et al., 1993), based on requirements for cattle [11]

### Mineral levels in hair samples of lactating cows

Hair samples collected during survey were analyzed for the same minerals as in feeds and forages. Mineral levels in hair must reflect the concentration and/or activity of the certain minerals in other parts of the body and reflect dietary mineral status of dairy cows [34]. The average levels of Cu and Zn in hair were 6.77 and 63.51 ppm, respectively (Table 6). When compared with critical levels for Cu (<10 ppm) and Zn (<100 ppm), 50 and 100% cows showed sub-normal levels in hair samples indicating their dietary deficiency. It has been demonstrated in several studies that concentration of Zn in hair is correlated with dietary Zn intake [35, 36]. Studies have shown the level of Zn in hair on normal diet to be 120–150 ppm in dairy cows [1]. The Se level of the hair of cattle is a useful indicator of both the Se deficiency and Se toxicity [37]. Most studies had shown that dairy cows with hair values consistently below 0.25 ppm probably need supplementation and that over 5 ppm may lead to clinical signs of selenosis [38]. The average Se level in hair samples was 3.48 ppm, indicating the adequacy of the element in the ration of dairy cows.

**Table 6:** Mineral content in hair samples of dairy cows

Particular	Ca (%)	P (%)	Mg (%)	S (%)	Na (%)	K (%)	Cu (ppm)	Zn (ppm)	Mn (ppm)	Se (ppm)
Hair samples (*n* = 20)	0.39 ±0.04	0.09 ±0.01	0.31 ±0.06	2.99 ±0.09	0.14 ±0.03	0.83 ±0.10	06.77 ±0.45	63.51 ±4.05	207.96 ±50.35	3.48 ±0.33

### Daily mineral intake by a lactating cow

The daily intake of different minerals by a HF crossbred cow (400 kg body weight) yielding 10 kg milk (4% fat), with the prevailing feeding system in the surveyed area is presented in Table 7. Since mineral mixture supplementation was not being followed, so the intake of minerals through feeds, wet forages and dry roughages with the 60% bio-availability [39, 40] was taken as index of total mineral supply and compared with the recommended requirements to know the dietary mineral adequacy/deficiency. Ration of dairy cows was found to be deficient in Ca, P, Mg, S, Cu, Zn and Co. Hence, it is necessary to supplement these minerals in the ration. It was observed that K, Na, Mn, Fe, Mo and Se in the ration of cows were found to be adequate. Supplementation of Cu and Zn in the form of chelates found to be more effective in curing problem of anestrous [41] and deficient trace minerals in the surveyed area may be supplemented in chelated form for better bio-availability and retention in the animal system.

**Table 7:** Minerals availability vis-à-vis requirement for dairy cow yielding 10 kg milk/day (4% fat)

Attributes	DMI (kg/d)	Ca (g)	P (g)	Mg (g)	S (g)	Cu (mg)	Zn (mg)	Co (mg)
Mineral requirement	11.50	48.10	30.80	23	23	115	920	5.75
Mineral availability								
Wheat bran (*Triticum aestivum*)	2.5	7.0	2.25	2.75	2.0	6.70	25.53	0.45
Rice straw (*Oryza sativa*)	4.0	12.8	3.20	4.64	4.4	11.96	107.12	1.44
Gram flour (*Cicer arietinum*)	1.0	1.20	3.70	1.50	1.90	7.68	35.60	0.29
Local grasses	2.0	12.80	7.20	5.60	3.60	21.74	87.01	0.72
Mustard cake (*Brassica campestris*)	1.0	6.25	10.0	2.60	9.25	7.56	48.89	0.39
Gram chunni (*Cicer arietinum*)	1.0	4.40	2.80	2.40	1.20	7.81	33.48	0.27
Daily mineral availability from traditional feeding	11.50	44.45	29.15	19.5	22.35	63.45	337.63	3.56
Degree of deficiency (%)		7.58	5.35	3.50	2.60	44.82	63.30	38.08

### Formulation of area specific mineral mixture

Information on the actual intake of each type of feeds and forage for a particular level of milk production was collected from each of the individual dairy farmer, to calculate intake of various mineral elements against the requirement. Total mineral intake from feeds and forages was compared against the requirements on dry matter basis (Table 8), to identify quantitative deficiency and adequacy of minerals. Based on the degree of deficiency, specification of mineral mixture used in the state was modified with area specific mineral mixture, by incorporating deficient minerals at higher levels and reducing or excluding excess minerals from the formulation, for supplementing the dairy cows in the Jharkhand state (Table 9). To enhance the usefulness of mineral mixture, chromium was also incorporated in the formulation.

**Table 8:** Mineral requirements for dairy cows

Particular	Calcium (Ca)	Phosphorus (P)
Maintenance (g)	16	11
Milk yield (g/kg)	3.21	1.98
Mg and S : 0.20% of DM intake	Copper : 10 ppm	Manganese : 40 ppm
Na : 0.18% of DM intake	Iron : 50 ppm	Cobalt : 0.50 ppm
K : 0.90% of DM intake	Zinc : 80 ppm	Selenium : 0.30 ppm
Cl : 0.25% of DM intake	Iodine : 0.60 ppm	Chromium: 0.5 ppm

**Table 9:** Area specific mineral mixture formulation for the state of Jharkhand

Sl. No.	Characteristic	Requirement
1.	Calcium (%), Min.	21.0
2.	Phosphorus (%), Min.	12.5
3.	Magnesium (%), Min.	3.0
4.	Sulphur (%), Min.	2.0
5.	Copper (%), Min.	0.20
6.	Zinc (%), Min.	1.40
7.	Cobalt (%), Min.	0.016
8.	Iodine (%), Min.	0.026
9.	Chromium (%), Min.	0.004

Note: Values for requirement at Sl. No. 1 to 9 are on dry matter basis

## Conclusions

It was evident from the present study that majority of the dairy cows in Jharkhand state were deficient in Ca, P, Mg, S, Cu, Zn and Co. Therefore, it is necessary to supplement these minerals in the ration of dairy cows by formulating area specific mineral mixture, having highly bio-available mineral salts. Deficient trace minerals, except Co, may be supplemented in the form of chelates, for better bio-availability and improving productivity, reproductive efficiency and productive life of dairy cows.

## Abbreviations

%: Per cent
ADF: Acid detergent fibre
AIA: Acid insoluble ash
Ca: Calcium
CF: Crude fibre
Co: Cobalt
CP: Crude protein
Cu: Copper
EE: Ether extract
Fe: Iron
INAPH: Information Network for Animal Productivity and Health
K: Potassium
Kg: Kilo gram
Mg: Magnesium
Mn: Manganese
Mo: Molybdenum
